# The role of continuous glucose monitoring (CGM) in psychiatric symptom management

**DOI:** 10.1017/S1092852925100540

**Published:** 2025-09-10

**Authors:** Melanie C. Zhang, Roger S. McIntyre

**Affiliations:** 1 Department of Psychiatry, University of Toronto, Toronto, ON, Canada; 2 Department of Pharmacology & Toxicology, University of Toronto, Toronto, ON, Canada

**Keywords:** Continuous glucose monitoring, major depressive disorder, generalized anxiety disorder, diabetes mellitus, blood glucose fluctuations

## Abstract

Continuous glucose monitoring (CGM) has revolutionized diabetes management by providing real-time data on blood glucose fluctuations. Unlike traditional methods, CGM systems offer continuous feedback, enabling individuals to better regulate glucose levels in response to lifestyle factors such as diet, exercise, and stress. This technology has been shown to improve glycemic control and stabilize HbA1c levels. Beyond its primary role in diabetes management, emerging research highlights the relationship between metabolic health and mental wellbeing. Glucose dysregulation has been implicated in mood instability, and fluctuations in blood glucose levels may directly influence emotional states. Notably, some researchers have proposed reclassifying major depressive disorder (MDD) as “Metabolic Syndrome Type II” due to shared pathophysiological mechanisms involving glucose homeostasis and inflammation. Given these connections, CGM technology may offer mental health benefits by promoting glucose stability. For individuals with diabetes who also experience psychiatric conditions such as MDD or generalized anxiety disorder (GAD), CGM use may contribute to improved mood regulation and reduced psychiatric symptoms. By addressing both metabolic and mental health concerns, CGM holds promise as a valuable tool in enhancing overall wellbeing. Further research is warranted to explore the full potential of CGM in supporting mental health outcomes in individuals with metabolic disorders.

## Introduction

Continuous glucose monitoring (CGM) has emerged as a pivotal tool in the management of diabetes mellitus, offering real-time data on blood glucose fluctuations. Unlike traditional glucose monitoring methods, such as finger-stick tests, CGMs provide continuous feedback on how various factors—such as diet, physical activity, sleep, stress, and insulin dosages—affect blood glucose levels. This real-time data empowers individuals to better regulate their blood glucose levels and adapt to fluctuations, contributing to improved overall glucose control and more stable HbA1c levels.

Simultaneously, in recent years, the significance of metabolic health in mental wellbeing has gained increasing attention, especially as research reveals a strong connection between metabolic disorders and psychiatric conditions. Dysregulation of glucose metabolism, for example, has been linked to mood instability, and fluctuations in blood glucose levels are known to have direct impacts on mood regulation. In fact, some researchers have proposed that major depressive disorder (MDD) be reclassified as “Metabolic Syndrome Type II” due to shared pathophysiological mechanisms involving metabolic networks, including insulin-glucose homeostasis and inflammatory processes.^1^

Given the clinical and pathophysiological overlap between metabolic and psychiatric health, it can be postulated that CGM technology, by stabilizing glucose levels, may offer additional benefits beyond glycemic control. Specifically, for individuals with diabetes who also experience psychiatric symptoms, such as MDD and GAD, CGM has the potential to help stabilize mood and reduce the risk of psychiatric symptoms, thereby enhancing both physical and mental wellbeing. This paper examines the potential relationship between CGM use, psychiatric symptom management, and overall quality of life in individuals with diabetes, as well as exploring the broader applications of CGMs for individuals without diabetes seeking alternative approaches to managing psychiatric symptoms.

## Mechanisms and evidence for CGM’s impact on mental health in individuals with diabetes

From a physiological point of view, it is known that both peripheral and central metabolic states play significant roles in the etiopathogenesis of MDD and other common psychiatric conditions, such as generalized anxiety disorder (GAD). In fact, high glycemic variability and persistent hyperglycemia have been associated with an increased incidence of MDD and GAD.^2^ Although the underlying mechanisms remain unclear, several prominent theories offer explanations. One theory posits that central and peripheral inflammation, mediated by cytokines released from adipocytes and immune cells, contributes to psychiatric symptoms in individuals with metabolic syndrome.^3^ In the same vein, Pistis et al.^1^ found that disturbances in metabolic networks—eg, insulin-glucose homeostasis, immuno-inflammatory processes, adipokine synthesis and secretion, intracellular signaling cascades, and mitochondrial respiration—are implicated in the pathophysiology, brain volumetric changes, symptomatic expression (eg, neurocognitive decline), and medical comorbidity in depressive disorders.

The central nervous system, similar to the pancreas, is a critical modulator of the metabolic milieu and is endangered by chronic abnormalities in metabolic processes. Others suggest hormonal disruptions, such as elevated cortisol levels or impaired insulin and glucagon-like peptide (GLP) function, may also play a role.^4^ Additionally, alterations in the gut microbiome and glial cell function may be involved, as metabolic disturbances can disrupt the gut-brain axis and impair neuroinflammation.^5^ While the underlying mechanisms continue to be investigated, one can hypothesize based on current evidence that stabilizing glucose levels through CGM use may improve mood, reduce diabetes-related distress, and potentially alleviate depressive symptoms, thereby decreasing the prevalence of psychiatric conditions, such as MDD and GAD.

The use of CGM has been shown to improve patient-reported outcomes in individuals with diabetes, with benefits likely stemming from both the reduction in illness management burden and the biological effects of physiological glucose stabilization.^6^ On one hand, CGMs reduce the daily hassles of diabetes management, including frequent finger pricks, manual glucose logging, and the anxiety associated with unpredictable blood sugar fluctuations. This reduction in management burden likely contributes to lower diabetes-related distress and improved quality of life, as shown by Ssemmondo et al.^6^ Moreover, growing evidence suggests that glucose stabilization itself provides biological benefits that positively impact mental health. For example, stable glucose levels reduce the formation of advanced glycation endproducts (AGEs), mitigate oxidative stress, and prevent inflammation—factors known to adversely affect brain function and mood regulation.^7^ Additionally, hyperglycemia and glycemic variability have been linked to increased pro-inflammatory cytokine release, which disrupts the blood–brain barrier and impairs neuroplasticity in brain regions involved in mood regulation, such as the hippocampus and prefrontal cortex.^7^

Metabolic disorders, such as obesity and diabetes mellitus (DM), may further affect brain cellular integrity and functional connectivity, potentially leading to cognitive and mood disturbances. This phenomenon has been termed “metaboptosis,” due to the finding that chronic metabolic dysregulation can lead to premature cellular aging and neuronal dysfunction, which may contribute to psychiatric conditions.^8^ The role of glucose in brain function, and by extension, poor mental health symptoms, is also well-documented. By maintaining tighter glucose control, CGMs may also help preserve neuronal and glial cell function, support healthy neurotransmitter signaling, and thus reduce the risk of mood disturbances. These effects are particularly relevant for individuals with metabolic disorders, where altered glucose metabolism exacerbates psychological symptoms.^9^

The bidirectional relationship between metabolic disorders, such as type 2 diabetes mellitus (T2DM), and mental health conditions like MDD is defined not only by shared biological mechanisms but also by how each condition exacerbates the other. It is important to recognize that this interaction extends beyond the biological pathways—such as inflammation, insulin resistance, and neuroplasticity impairment—since each disorder can influence the severity and progression of the other. For instance, metabolic dysregulation in diabetes can worsen mood and cognitive function, while the emotional distress and psychological stress associated with MDD can destabilize glucose regulation, creating a feedback loop that complicates both management and treatment. This bidirectional nature underscores the importance of innovative therapeutic interventions, such as CGM, which provide an opportunity to address both conditions simultaneously. By breaking the cycle of metabolic and psychiatric exacerbation, CGMs can play a pivotal role in integrated treatment approaches, enhancing overall patient outcomes and offering a more holistic solution to the comorbidity between metabolic and psychiatric disorders.

As previously discussed, the association between T2DM and MDD is mediated by both shared biological mechanisms (e.g., inflammatory pathways). Dysregulated blood glucose, particularly hyperglycemia, triggers a cascade of damaging effects that impact both metabolic and psychiatric health. Hyperglycemia promotes the formation of AGEs, which activate receptors for AGEs (RAGE), leading to systemic inflammation and the release of pro-inflammatory cytokines such as IL-6, TNF-α, and C-reactive protein (CRP). This inflammatory response increases oxidative stress by raising the production of reactive oxygen species (ROS) and depleting antioxidant defenses, which in turn damages cellular structures, including lipids, proteins, and DNA. In the brain, these processes disrupt neuroplasticity, impair brain-derived neurotrophic factor (BDNF) signaling, and alter mood regulation.^1^^,^^7^

Furthermore, hyperglycemia-induced oxidative stress and inflammation contribute to endothelial dysfunction, compromising the blood–brain barrier and allowing inflammatory mediators to infiltrate the central nervous system.^10^ This cascade activates microglia, amplifying neuroinflammation and exacerbating mood disturbances, thus creating a vicious cycle where metabolic dysregulation worsens psychiatric symptoms and vice versa.

Conversely, chronic psychological stress and trauma activate the body’s “fight or flight” response, which disrupts immune function and the gut microbiome, further impairing glucose regulation.^10^ The overlapping biological mechanisms between mental health and metabolic dysregulation—such as inflammation, insulin resistance, and neuroplasticity impairment—form a reciprocal relationship where each condition exacerbates the other. This bidirectional dynamic highlights the complexity of managing comorbid T2DM and psychiatric disorders like MDD. For instance, while metabolic dysfunction in T2DM worsens mood and cognitive function, the emotional stress associated with MDD can destabilize glucose regulation, complicating both management and treatment.^11^

The aforementioned shared biological mechanisms are further supported by research identifying common genetic and molecular pathways. Liu et al.^12^ demonstrated that both T2DM and MDD involve dysregulation in immune system signaling, tau protein formation, and neuronal apoptosis. Both conditions also exhibit hormonal imbalances, such as hypercortisolemia and insulin resistance, autonomic nervous system dysfunction, systemic inflammation, and disruptions in the gut-brain axis—all contributing to neurodegeneration and impaired emotional regulation. Farzi et al.^13^ highlighted the role of oxidative stress and mitochondrial dysfunction in these processes, further underlining the biological overlap between T2DM and MDD. The co-occurrence of these disorders elevates the risk of cardiovascular complications and other comorbidities, as emphasized by Subba et al.^14^, reinforcing the need for integrated treatment approaches.

In this context, CGM presents a promising tool for addressing both T2DM and MDD simultaneously. By providing real-time data on glucose fluctuations, CGMs offer valuable insights into the metabolic processes that contribute to psychiatric symptoms. By stabilizing glucose levels, CGMs not only improve metabolic control but also help mitigate mood disturbances, cognitive impairments, and emotional dysregulation commonly seen in individuals with both T2DM and MDD. Notably, CGMs reduce the burden of diabetes management, eliminating the need for frequent finger pricks and manual glucose logging, which alleviates diabetes-related distress and improves quality of life.^6^ This stabilization of glucose levels can reduce the anxiety and stress associated with unpredictable blood sugar fluctuations, which in turn may lead to improved mental health outcomes. CGMs could be especially beneficial for individuals with trauma-related disorders, who are at increased risk for developing T2DM due to shared biological mechanisms.^5^ Ultimately, CGMs offer an integrated, non-pharmacological approach to managing the comorbidities of metabolic and psychiatric health, targeting both the metabolic and psychological aspects of these disorders and providing a holistic solution that may improve overall patient well-being.

CGM offers several potential benefits for psychiatric symptom management beyond the traditional approaches used in diabetes care. By allowing for real-time stabilization of glucose levels, especially when paired with an insulin pump, CGMs help individuals approximate the physiological glucose homeostasis seen in individuals without diabetes. One can postulate that the greater alignment of glucose levels with normal physiological ranges could lead to reductions in mood disturbances and improvements in mental health outcomes from a psychological and physiological perspective.

A summary of key clinical findings is provided in the table below.

As seen in Table 1, Hermanns et al.^15^ found that elevated depression and diabetes-related distress were significantly associated with higher glucose levels. The authors suggested that personalized symptom profiles informed by glucose data could improve the precision of diabetes symptom monitoring, ultimately enhancing mental health outcomes. Similarly, Ssemmondo et al.^6^ demonstrated that intermittently scanned CGM (isCGM) significantly reduced diabetes-related distress and improved HbA1c levels in individuals with high baseline levels of distress. These findings further indicate that CGM can address both the psychological burden of diabetes management and its physiological manifestations, potentially reducing psychiatric symptoms and improving overall wellbeing.

**Table 1.** Summary of Clinical Evidence Supporting the Mental Health and Psychosocial Benefits of Continuous Glucose Monitoring

References	Population	Intervention/Technology	Key findings
Hermanns et al.^14^	Participants with diabetes	CGM and Ecological Momentary Assessment	Elevated depression and diabetes-related distress associated with higher glucose levels; suggests CGM can improve precision of symptom monitoring.
Ssemmondo et al.^6^	Participants with diabetes and high baseline distress	Intermittently scanned CGM (isCGM)	Significantly reduced diabetes-related distress and improved HbA1c levels.
Polonsky et al.^15^	Adults with Type 1 diabetes	CGM (DIAMOND randomized trial)	Significant improvements in diabetes-specific quality of life, including reductions in diabetes distress and hypoglycemic fear, with increased hypoglycemic confidence.
Gilbert et al.^16^	Participants with insulin-treated diabetes	Real-time CGM (rtCGM)	Showed improvements in HbA1c and quality of life.
Soriano and Polonsky^17^	Adults with insulin-using type 2 diabetes	Real-time CGM (rtCGM)	Associated with significant improvements in psychosocial outcomes.

Polonsky et al.^16^ reported that CGM use led to significant improvements in diabetes-specific quality of life, including reductions in diabetes distress, hypoglycemic fear, and greater hypoglycemic confidence. The ability to reduce these anxieties associated with blood sugar fluctuations may not only improve physical outcomes but also enhance emotional stability.^19^ These early findings provide preliminary support that CGM use can reduce both the psychological and physical burdens of diabetes management, improving overall wellbeing and reducing psychiatric symptoms.

Additionally, as previously mentioned, metabolic dysregulation is a key feature of, and clinical predictor for, mood-, stress-, and trauma-related disorders such as MDD, GAD, and post-traumatic stress disorder. Recent evidence supports the notion that fluctuations in blood sugar levels may be associated with psychiatric symptoms, suggesting that metabolic data—such as that collected through CGM—could provide valuable insights into the mind-body connection. For instance, Hermanns et al.^20^ reported that hyperglycemia values were negatively correlated with positive mood ratings and were associated with more negative mood ratings, suggesting that stabilization of blood glucose levels could improve mood by mitigating the intensity of glucose-related mood fluctuations.

It then stands to reason that individuals with diabetes, who regularly experience blood sugar fluctuations, may benefit from CGM not only for managing glycemic control but also for enhancing mental health on a psychological level. The use of CGM can help individuals better understand the relationship between physiological states and psychological well-being, offering real-time insights into how fluctuations in glucose levels correlate with mood and emotional regulation, in addition to the potential direct physiological benefits discussed previously. These include the aforementioned pathways of reducing systemic inflammation, oxidative stress, and the formation of AGEs—all of which are known to adversely affect brain function and mood regulation.

The psychological benefits of CGM are likely inextricably linked with the biological advantages of more stable glucose control. By reducing both the emotional distress associated with diabetes management and the physical consequences of dysregulated glucose, CGMs provide a holistic approach to improving patient well-being. From a patient perspective, the ability to more precisely track and manage glucose levels not only alleviates anxieties such as hypoglycemic fear but also fosters a greater sense of control over one’s health, leading to improved quality of life and satisfaction with care. Moreover, from a physiological standpoint, CGMs help approximate normal physiological glucose homeostasis, a key factor in mitigating the systemic inflammation, oxidative stress, and neuroplasticity impairments that contribute to psychiatric symptoms. This dual benefit—enhancing mental health while stabilizing metabolic function—supports a more comprehensive, patient-centered approach to diabetes care, emphasizing the importance of integrated treatment strategies that consider both the body and mind. By aligning with normal glucose regulation, CGMs could not only provide an effective tool for managing diabetes but also offer a powerful means of improving mental health outcomes, ultimately benefiting patients on multiple levels.

## Applications of CGM in non-diabetic populations and limitations

While CGMs are primarily used by individuals with diabetes, they may also benefit those without diabetes who are interested in a personalized approach to wellness, for example, providing the function of reducing baseline distress or improving quality of life. By offering insights into an individual’s unique metabolic responses, CGMs may provide valuable data for customizing dietary and lifestyle changes, potentially alleviating psychiatric symptoms and offering additional solutions for mental health management in those with and without the formal diagnosis of a metabolic condition.

However, while CGMs offer many benefits, they are not without limitations, particularly for non-diabetic use. One major barrier is the potential need for a prescription from healthcare providers, which can make CGMs inaccessible to individuals without insurance or those in lower socioeconomic brackets. Walker et al.^21^ found significant disparities in CGM use based on socioeconomic status, race, and ethnicity, highlighting inequities in healthcare access. For example, uninsured individuals and those from minority ethnic backgrounds were found to have lower odds of receiving CGM prescriptions.^22^ Additionally, although CGMs are often covered by insurance for individuals with diabetes, they are not typically covered for those with prediabetes or those seeking to use CGMs for general wellness monitoring. This creates a barrier to access for those who may benefit from CGM use but do not meet the clinical criteria for coverage. Moreover, there is limited research on the optimal glucose target ranges for individuals without diabetes, which raises concerns about the potential effectiveness and safety of CGMs outside a clinical context.^23^

It ‘is also important to note that CGMs cannot determine the root cause of the underlying symptoms. While they can provide real-time glucose data, the physiological factors contributing to psychiatric symptoms, such as inflammation, oxidative stress, or hormonal dysregulation, require a more comprehensive clinical approach to fully understand.

## Conclusion

CGM offers significant potential for improving psychiatric symptom management by enhancing glycemic control and reducing diabetes-related distress. By stabilizing glucose levels and minimizing the burden of diabetes management, CGMs can help improve mood and alleviate psychiatric symptoms, such as depression and anxiety. Studies have demonstrated that CGM use leads to improved HbA1c levels, reduced diabetes distress, and better overall wellbeing. Furthermore, the data provided by CGMs can help uncover patterns between metabolic fluctuations and psychological wellbeing, empowering individuals to take a more active role in managing both their physical and mental health.

The bidirectional relationship between glucose metabolism and mental health suggests that CGM technology could have far-reaching implications—not only for diabetes management but also for broader applications in personalized health. Continued research into the integration of metabolic and mental health will help further establish CGM as a powerful tool in personalized medicine, offering new insights and potential solutions for both physical and mental wellbeing.

## Data Availability

No datasets were generated or analyzed during the current study.
